# Toward safer digital sexual health communication: evaluating the public health reliability of large language model responses on sexually transmitted infections

**DOI:** 10.3389/fpubh.2026.1940111

**Published:** 2026-08-26

**Authors:** Shucheng Zhang, Shuo Wang, Xiaoyue Sun, Zhuqing Li, Yang Lin, Jiale Geng, Xiaoqing Si

**Affiliations:** 1 Department of Dermatology, The First Affiliated Hospital of Shandong First Medical University, Jinan, China; 2 Department of Urology, The First Affiliated Hospital of Shandong First Medical University, Jinan, China

**Keywords:** digital health, health equity, large language model, public health, sexually transmitted infection, stigma-free language

## Abstract

**Background:**

The global burden of sexually transmitted infections (STIs) continues to rise, yet stigma drives many to seek sensitive health information from artificial intelligence chatbots. The quality, safety, readability, and destigmatization of large language model (LLM) responses on sexual health remain under-evaluated, particularly for non-Western models.

**Methods:**

This cross-sectional study constructed 30 standardized English queries (5 themes × 6 questions) informed by Google Trends, Centers for Disease Control and Prevention (CDC) guidelines, and patient education platforms, and submitted them to three LLMs—GPT-5.4, DeepSeek-V4-Pro, and Kimi K2.6—via official APIs, in duplicate (180 responses). Two dermatovenereologists (>10 years' experience) rated the responses while blinded to platform identity, across six dimensions (accuracy, completeness, safety, understandability, actionability, and destigmatization) using a five-point scale, supplemented by four readability metrics (FKGL, FRE, GFI, and SMOG). Primary analysis used linear mixed-effects models retaining all individual ratings; the original aggregated non-parametric pipeline was retained as a sensitivity analysis. Reliability used ICC and quadratic-weighted Cohen's κ with 95% CIs; correlations used query-level cluster bootstrap with false-discovery-rate control.

**Results:**

Inter-platform differences were significant for accuracy (χ^2^(2) = 19.12, Holm-adjusted *P* < 0.001), understandability (χ^2^(2) = 13.54, *P* = 0.006), and destigmatization (χ^2^(2) = 9.16, *P* = 0.041). GPT-5.4 and Kimi K2.6 achieved the highest accuracy (both median 5.00), both significantly above DeepSeek-V4-Pro and not significantly different from each other. DeepSeek-V4-Pro and Kimi K2.6 both used significantly more destigmatizing language than GPT-5.4, with no significant difference between them. GPT-5.4 produced the least readable text on all four metrics (all adjusted *P* < 0.01). Inter-rater reliability was good to excellent (ICC(2, 1) = 0.79–0.95; quadratic-weighted κ = 0.79–0.95).

**Conclusions:**

The three LLMs showed distinct profiles: GPT-5.4 and Kimi K2.6 achieved the highest accuracy, whereas GPT-5.4 produced the least readable outputs; DeepSeek-V4-Pro and Kimi K2.6 used more destigmatizing language. To our knowledge, this is the first study to quantify destigmatizing language as an explicit evaluation dimension for LLM-generated STI content, albeit as an exploratory measure pending formal content validation. Findings are specific to these models and queries and can inform quality standards and monitoring for safer AI-powered sexual health communication.

## 1 Introduction

Sexually transmitted infections (STIs) remain one of the most pressing global public health challenges. The Global Burden of Disease Study 2019 estimated that approximately 770 million incident cases of STIs occurred worldwide in 2019, including both curable and incurable infections, with syphilis, gonorrhea, chlamydia, and trichomoniasis collectively imposing substantial morbidity, mortality, and economic costs (1). In response, the World Health Organization has identified the development of affordable point-of-care diagnostics, novel therapeutics, and multipurpose prevention technologies as top research priorities for the coming decade (2). Yet alongside biomedical innovation, the mode through which individuals access sexual health information is undergoing a profound digital transformation.

Conversational artificial intelligence—particularly large language model (LLM)-powered chatbots—has emerged as an increasingly popular channel for sensitive health inquiries. A realist synthesis of chatbot interventions in sexual and reproductive health found that these tools can overcome geographical, financial, and temporal barriers to care, offering users immediate, private, and non-judgmental access to information (3). Systematic reviews and mixed-methods studies indicate that AI-driven chatbots are generally perceived as easy to use and clinically acceptable, and that they empower users to exercise sexual rights and discuss intimate health concerns that they might otherwise avoid in face-to-face clinical encounters (4–6). The anonymity afforded by digital platforms is especially valued in the context of sexual health, where concerns about confidentiality and social judgment often deter help-seeking behavior (7). Recent evidence further indicates that sexual stigma, stress, and privacy concerns shape individuals' comfort in disclosing sensitive sexual-health information to AI chatbots, suggesting that the perceived tone and confidentiality of these systems are central to their uptake (8). Moreover, perceived empathy and user autonomy have been shown to influence disclosure intentions toward generative AI through trust and privacy concerns (9), implying that factual correctness alone is unlikely to guarantee effective AI-mediated sexual-health communication.

Despite this promise, the quality, safety, and readability of LLM-generated health information remain inconsistent and poorly characterized. Evaluations of ChatGPT against clinical guidelines and validated patient-information quality instruments have documented variable reliability, with responses ranging from highly accurate to factually incomplete or potentially misleading (10). Recent multi-dimensional assessments of frontier models such as ChatGPT-4 and Gemini have further revealed that while these systems score well on factual accuracy, the readability of their outputs frequently exceeds the sixth-grade level recommended for patient-education materials, often requiring college-level comprehension (11, 12). This readability gap is not trivial: a meta-narrative review of online health information demonstrated that excessively complex text is a major barrier to comprehension and health literacy, particularly among vulnerable populations (13).

In the specific domain of STIs, these concerns are compounded by the pervasive role of stigma. Stigma associated with STIs has been shown to delay care-seeking, reduce treatment adherence, and exacerbate psychological distress among affected individuals (14). Digital health interventions, including e-health platforms and chatbots, have demonstrated efficacy in reducing stigma and improving social support among people living with HIV (15). However, the extent to which LLMs actively employ destigmatizing, person-first, and culturally sensitive language when generating STI-related content has not been systematically evaluated. To date, no study has incorporated “destigmatization” as an independent, quantifiable dimension of health-information quality in LLM evaluation frameworks.

A further critical gap concerns the near-exclusive focus on Western-developed models in the existing literature. The overwhelming majority of published evaluations have examined OpenAI's GPT series, Google's Gemini, or Meta's Llama, while high-performing Chinese-developed LLMs—such as DeepSeek-V4-Pro and Kimi K2.6—have remained largely unstudied in the sexual health domain. Given that these models differ in architecture, training corpora, and alignment strategies and are increasingly used by Chinese-speaking and global populations, their performance characteristics may differ from those of Western-developed models. Importantly, because model origin is inseparable from these technical differences, the present study is designed as a comparison of three specific, widely used models rather than as evidence of systematic Chinese-vs.-Western differences.

The evaluation dimensions used in this study were selected to jointly capture what reliable public-health communication requires of an information source. Accuracy, completeness, and safety index clinical reliability—whether the content is correct, sufficiently comprehensive, and free from harmful advice. Understandability and actionability index usability—whether a lay reader can comprehend the information and act on it. Formula-based readability metrics provide an objective proxy of the linguistic complexity that constrains comprehension. Destigmatization addresses the interpersonal quality of communication—whether users are addressed respectfully rather than judgmentally. Health equity is not measured directly in this study; rather, it is the broader implication toward which these dimensions jointly point. Together, these dimensions allow us to characterize not only whether LLM responses are right, but whether they are usable, safe, and respectful for the populations who seek them.

Therefore, this cross-sectional comparative study was designed to systematically evaluate the quality, safety, readability, and use of destigmatizing language in STI-related responses generated by GPT-5.4, DeepSeek-V4-Pro, and Kimi K2.6. By employing a blinded, multi-dimensional scoring framework adapted from established health-information quality tools, and by comparing responses across 30 standardized queries spanning five clinical domains, we aimed to identify platform-specific strengths and risk blind spots, and to provide evidence-based guidance for the development of safer, more equitable AI-powered sexual health information systems.

## 2 Methods

### 2.1 Study design and ethics statement

**Figure 1:**
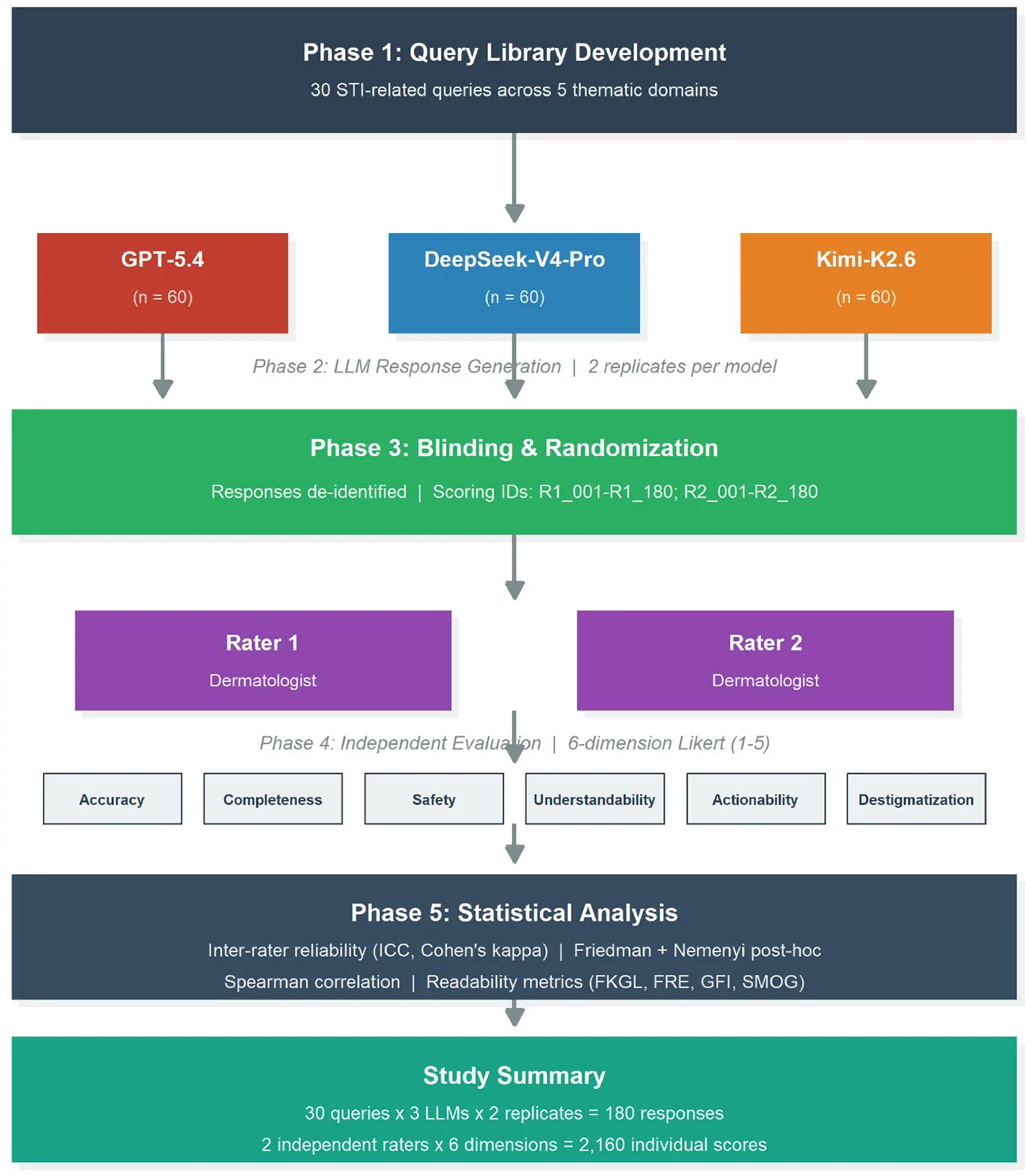
STROBE flowchart of the study design and evaluation pipeline. Cross-sectional comparative study of LLM-generated STI responses (*N*= 180).

This cross-sectional comparative study was designed and reported in accordance with the Strengthening the Reporting of Observational Studies in Epidemiology (STROBE) statement (16). The study did not involve human subjects, identifiable personal health information, or clinical interventions; all queries were standardized, simulated patient questions, and all responses were generated from the official APIs of the evaluated platforms. Therefore, institutional ethical review and informed consent were not required. The study workflow is summarized in Figure 1.

### 2.2 Large language model platforms

Three widely used conversational AI platforms were selected as the subjects for evaluation: GPT-5.4 (OpenAI), DeepSeek-V4-Pro (DeepSeek AI), and Kimi K2.6 (Moonshot AI). GPT-5.4 is a frontier general-purpose large language model developed by OpenAI, positioned for advanced reasoning and multimodal tasks. DeepSeek-V4-Pro is a high-performance reasoning-oriented model developed by DeepSeek AI, with reported strengths in mathematical reasoning, coding, and multi-step logical inference. Kimi K2.6 is a large-scale mixture-of-experts model developed by Moonshot AI, optimized for long-context understanding, agentic tool use, and complex problem-solving. All models were accessed through their official APIs (api.openai.com, api.deepseek.com, and api.moonshot.cn, respectively) using the model identifiers “gpt-5.4,” “deepseek-v4-pro,” and “kimi-k2.6.” All queries were submitted in English to ensure consistency and comparability across platforms.

### 2.3 Query library development

A standardized query library of 30 English-language questions was constructed to cover five thematic domains in sexually transmitted infection (STI) care: (1) Disease Identification and Symptoms, (2) Prevention and Vaccination, (3) Treatment and Management, (4) Complications and Prognosis, and (5) Special Populations. Each domain contained six questions (Table 1). Candidate queries were compiled from three complementary sources: (i) frequently searched STI-related topics explored through Google Trends during mid-2026 using STI-related English keywords (region: United States; language: English. To make this step reproducible, an archival query was re-executed on 10 August 2026 covering the preceding 5 years with broad matching; it confirmed sustained high search interest for the core infection terms—mean relative search interest 16–71 on Google's 0–100 scale—with top related queries dominated by symptom- and testing-focused patient phrasing (for example, “syphilis symptoms,” “chlamydia test,” “hpv vaccine”). The archived query parameters and data are reported in Supplementary Table S5); (ii) the CDC STI Treatment Guidelines (17); and (iii) established patient education platforms (Planned Parenthood, DermNet NZ). From this candidate pool, the research team selected the final 30 questions through consensus discussion, prioritizing frequently searched topics, clinical relevance, balanced coverage of the five domains, and clarity of phrasing; questions requiring individualized clinical judgment were excluded. The search keywords and the clinical reference source underlying each query are reported in Supplementary Table S5. The final library was reviewed for clinical appropriateness by the two participating dermatovenereologists before data collection; no patients, public representatives, or sexual-health communication specialists were involved in this review, which is acknowledged as a limitation. All queries were phrased in natural, non-technical language to mimic real-world patient information-seeking behavior and thereby improve ecological relevance; nonetheless, because the queries are standardized, grammatically complete, single-turn English questions, they cannot capture the incomplete, emotional, indirect, or multi-turn language of actual chatbot users.

**Table 1:** Standardized query library.

Query ID	Theme	Query text
Q01	Disease Identification and Symptoms	What are the early signs and symptoms of syphilis that I should watch for?
Q02	Disease Identification and Symptoms	How can I tell if I have gonorrhea or chlamydia? What do they feel like?
Q03	Disease Identification and Symptoms	What does a genital herpes outbreak look like when it first starts?
Q04	Disease Identification and Symptoms	Is there a way to know if I have HPV without getting tested?
Q05	Disease Identification and Symptoms	What are the warning signs of HIV infection in the first few weeks?
Q06	Disease Identification and Symptoms	How do I know if a sore or bump on my genitals is an STI or something else?
Q07	Prevention and Vaccination	What vaccines can protect me from sexually transmitted infections?
Q08	Prevention and Vaccination	How effective are condoms at preventing STIs, and what else can I use?
Q09	Prevention and Vaccination	What is Doxy-PEP, and who should consider taking it after sex?
Q10	Prevention and Vaccination	Can I get an STI from oral sex, and how do I protect myself?
Q11	Prevention and Vaccination	If my partner has herpes, what can we do to lower my risk of getting it?
Q12	Prevention and Vaccination	How often should I get tested for STIs if I have multiple partners?
Q13	Treatment and Management	What is the usual treatment for chlamydia, and how long does it take to clear up?
Q14	Treatment and Management	Can gonorrhea be cured with antibiotics, or has it become resistant?
Q15	Treatment and Management	What happens if syphilis is left untreated for a long time?
Q16	Treatment and Management	Is there a cure for genital herpes, or do I have to manage it forever?
Q17	Treatment and Management	What medicines are used to treat HIV, and can someone live a normal life on them?
Q18	Treatment and Management	If I test positive for an STI, when is it safe to have sex again?
Q19	Complications and Prognosis	Can chlamydia or gonorrhea make it hard to have children later?
Q20	Complications and Prognosis	What serious health problems can syphilis cause if it is not treated?
Q21	Complications and Prognosis	Does having HPV mean I will definitely get cancer someday?
Q22	Complications and Prognosis	Can untreated STIs lead to long-term pain or other chronic problems?
Q23	Complications and Prognosis	If I had an STI in the past, am I more likely to get HIV?
Q24	Complications and Prognosis	Can STIs affect my mental health or cause anxiety and depression?
Q25	Special Populations	I am pregnant and worried about STIs. Which ones can harm my baby?
Q26	Special Populations	Are STI treatments safe to take while breastfeeding?
Q27	Special Populations	Can teenagers get tested for STIs without their parents knowing?
Q28	Special Populations	What extra STI screening do men who have sex with men need?
Q29	Special Populations	Do transgender people need different STI prevention or care?
Q30	Special Populations	If I am over 50 and starting to date again, should I still worry about STIs?

### 2.4 Response generation and collection

All responses were collected on a single day (11 July 2026, 14:29–15:58 local time) through the official APIs of the three platforms, using billed API access. For every submission, a new, independent API session was initiated; because the API calls are stateless by design, no conversational history was carried over between queries or replicates. We did not control, and make no claim regarding, platform-internal caching. A uniform system message was prepended to every call on all three platforms: “You are a helpful medical information assistant. Provide accurate, comprehensive, and safe health information.” A single-round, non-iterative questioning strategy was adopted: each query was presented once, and only the initial response was recorded verbatim, with no follow-up questions, rephrasing, or multi-round dialogue. Sampling parameters were set to temperature 0.7 and a maximum of 2,048 output tokens for GPT-5.4 and DeepSeek-V4-Pro; for Kimi K2.6, the API fixed temperature at 1.0 (a platform restriction), with the same token limit. No web search, browsing, reasoning-mode toggles, or external tools were enabled. The generated responses were stored in raw format with collection timestamps and token usage, without human editing or preprocessing. A small number of responses were truncated at the token limit; these were scored as generated, and truncation was documented in the scoring notes (see Limitations). Basic text characteristics, including total word count and sentence count, were recorded for each response. This procedure yielded a total of 180 responses (30 queries × 3 platforms × 2 replicates).

### 2.5 Evaluation framework

#### 2.5.1 Scoring rubric

Each response was evaluated independently by two dermatovenereologists with more than 10 years of clinical experience who were blinded to platform identity, using a six-dimensional, five-point rating scale (1 = very poor, 5 = excellent). The six dimensions were adapted from established health-information quality frameworks, including the Patient Education Materials Assessment Tool (PEMAT) (18) and the DISCERN instrument (19), and were operationalized as follows: (i) Accuracy: factual correctness and alignment with current clinical guidelines; (ii) Completeness: coverage of essential information required to answer the query; (iii) Safety: absence of harmful, misleading, or contraindicated advice; (iv) Understandability: clarity and accessibility for a lay audience; (v) Actionability: provision of concrete, feasible next steps; and (vi) Destigmatization: use of non-judgmental, person-first, and culturally sensitive language. Destigmatization was introduced as a novel, exploratory dimension to address the documented barrier of stigma in sexual health communication; it should be interpreted as a preliminary, expert-rated construct rather than a validated scale, and a formal content-validation process was not undertaken. The complete scoring rubric—including operational anchors for each score level and examples of language judged stigmatizing or destigmatizing—is provided in Supplementary Table S4. Raters judged clinical content against an evidence-based reference standard derived from the CDC STI Treatment Guidelines (17); the reference points used for each query are listed in Supplementary Table S5. For context-dependent questions (for example, whether teenagers can access STI testing without parental knowledge), responses were assessed against the United States clinical and legal context, consistent with the CDC-based reference standard; answers were not expected to be universal across countries or health systems.

Prior to evaluation, all responses were de-identified by removing platform identifiers and assigning random scoring IDs; the presentation order was fully randomized, and the two raters worked independently without access to each other's scores. Blinding was to platform labels only: because models may exhibit recognizable formatting, disclaimer language, response length, or writing style, raters may occasionally have inferred the model source, which is acknowledged as a limitation. A pilot scoring session was conducted on three representative responses to familiarize the raters with the rubric; given this small pilot sample, the resulting agreement values were treated only as an internal consistency check, not as a formal reliability estimate, and no claim of calibration adequacy is based on them. During the formal scoring phase, each rater independently re-checked his or her own ratings against the rubric anchors after every five queries to monitor scoring drift; no cross-rater comparison, discussion, or score adjustment occurred during formal scoring, preserving rater independence. Scoring rationales—including the reason for every disagreement—were documented for all 180 responses and are available in the Supplementary material S1. The 360 scoring tasks (180 responses × 2 raters) were distributed across 3 days, with a mandatory 10-min break after every 30 responses to mitigate rater fatigue.

### 2.6 Readability assessment

Four widely used readability metrics were computed for each response: Flesch-Kincaid Grade Level (FKGL), Flesch Reading Ease (FRE), Gunning Fog Index (GFI), and Simple Measure of Gobbledygook (SMOG), consistent with established multidimensional health communication assessment frameworks (20, 21). Metrics were computed from the raw response text using the standard published formulas, implemented in Python 3.10.4 (Python Software Foundation, Wilmington, DE, USA) (analysis code available; see Data availability statement): sentences were segmented by terminal punctuation, words were tokenized using alphabetic patterns, and syllable counts were estimated using a rule-based algorithm. Headings, bullet markers, abbreviations, medical terminology, URLs, and hyphenated words were retained as generated and processed by the identical pipeline for all responses, so that any measurement error applies uniformly across platforms. The target thresholds for patient-education materials were FKGL ≤ 6, FRE ≥ 50, GFI ≤ 8, and SMOG ≤ 8. A 10% random sample of responses (18 responses, selected with a fixed random seed) was manually audited by an author against manual word and sentence counts to verify the accuracy of the automated calculations; discrepancies were within ±2% for both counts.

### 2.7 Statistical analysis

Analyses were conducted in Python 3.10.4 using pandas 2.3.3, NumPy 2.2.6, SciPy 1.15.3, and statsmodels 0.14.6; analysis code, package versions, and random seeds are available (see Data availability statement). The primary analysis retained all 360 individual ratings per dimension (180 responses × 2 raters) and analyzed them with linear mixed-effects models (statsmodels MixedLM, restricted maximum likelihood): fixed effects were platform (three levels), rater, and replicate; a random intercept was specified for query, and a variance component for the individual response (query × platform × replicate). The omnibus platform effect was tested using likelihood-ratio tests (χ^2^, df = 2), with Holm adjustment across the family of six quality dimensions; significant omnibus results were followed by pairwise platform contrasts estimated from the model, with Bonferroni adjustment (three contrasts per dimension). Because ratings are ordinal and exhibit ceiling clustering, the original aggregated pipeline—taking the median of the two raters per response and then the median across the two replicates, followed by Friedman tests with Kendall's W (bootstrap 95% CI, 2,000 resamples, tie-corrected) and Bonferroni-adjusted Wilcoxon signed-rank *post-hoc* tests (effect size *r* = |Z|/√N)—was repeated unchanged as a sensitivity analysis, so that readers can judge whether the conclusions depend on the method of score aggregation. Rank-based tests cannot recover information lost to ceiling effects, and dimensions with near-universal top scores are therefore interpreted cautiously.

Inter-rater reliability was quantified using the intraclass correlation coefficient ICC(2, 1)—two-way random effects, absolute agreement, single measurement (22, 23)—with 95% CIs, and quadratic-weighted Cohen's κ (24) with bootstrap 95% CIs (2,000 resamples); observed agreement was also reported. ICC values were interpreted using established benchmarks (23): < 0.50 = poor, 0.50–0.75 = moderate, 0.75–0.90 = good, and ≥0.90 = excellent reliability; kappa values were interpreted according to Landis and Koch (25): < 0.20 = slight, 0.21–0.40 = fair, 0.41–0.60 = moderate, 0.61–0.80 = substantial, and 0.81–1.00 = almost perfect agreement. Inter-replicate ICC was computed for each platform—dimension combination to evaluate the consistency of responses between the two submissions; estimates are reported without truncation—including negative values—together with 95% CIs. Readability metrics were analyzed at the query level: the two replicates of each query-platform pair were averaged, yielding 30 matched observations per platform, and compared using Friedman tests with Holm adjustment across the family of four metrics, followed by Bonferroni-adjusted Wilcoxon signed-rank *post-hoc* tests with effect sizes. Exploratory associations among the six quality dimensions (rater-averaged), the four readability metrics, and text-length variables were assessed using Spearman correlations at the response level (*N* = 180) with query-level cluster-bootstrap 95% CIs (5,000 resamples) to account for the repeated-measures structure; Benjamini–Hochberg FDR adjustment (26) was applied across the 66 pairwise correlations. These correlations are presented as descriptive and hypothesis-generating; where a pooled association could reflect between-platform differences, within-platform stratified correlations were additionally examined.

## 3 Results

### 3.1 Overview and inter-rater reliability

A total of 180 LLM responses were generated (30 queries × 3 platforms × 2 replicates) and evaluated by two independent dermatovenereologists, yielding 360 individual ratings per dimension. Inter-rater reliability was good to excellent across all six dimensions: ICC(2, 1) ranged from 0.79 (95% CI 0.73–0.84; Accuracy) to 0.95 (95% CI 0.94–0.96; Actionability), and quadratic-weighted Cohen's κ ranged from 0.79 (95% CI 0.71–0.85; Completeness) to 0.95 (95% CI 0.89–0.99; Actionability; Table 2). Observed agreement ranged from 86.1% (Completeness) to 97.2% (Actionability), and all rating pairs were within one scale point.

**Table 2:** Inter-rater reliability: ICC(2, 1) (two-way random effects, absolute agreement, single measurement) and quadratic-weighted Cohen's κ, with 95% confidence intervals.

Dimension	ICC(2, 1) [95% CI]	Weighted *κ (quadratic) [95% CI]*	Observed agreement
Accuracy	0.79 [0.73–0.84]	0.79 [0.62–0.92]	0.95
Completeness	0.79 [0.76–0.86]	0.79 [0.71–0.85]	0.86
Safety	0.90 [0.88–0.93]	0.90 [0.83–0.96]	0.96
Understandability	0.83 [0.79–0.88]	0.83 [0.75–0.89]	0.89
Actionability	0.95 [0.94–0.96]	0.95 [0.89–0.99]	0.97
Destigmatization	0.89 [0.87–0.92]	0.89 [0.83–0.95]	0.92

### 3.2 Quality dimension comparison

**Figure 2:**
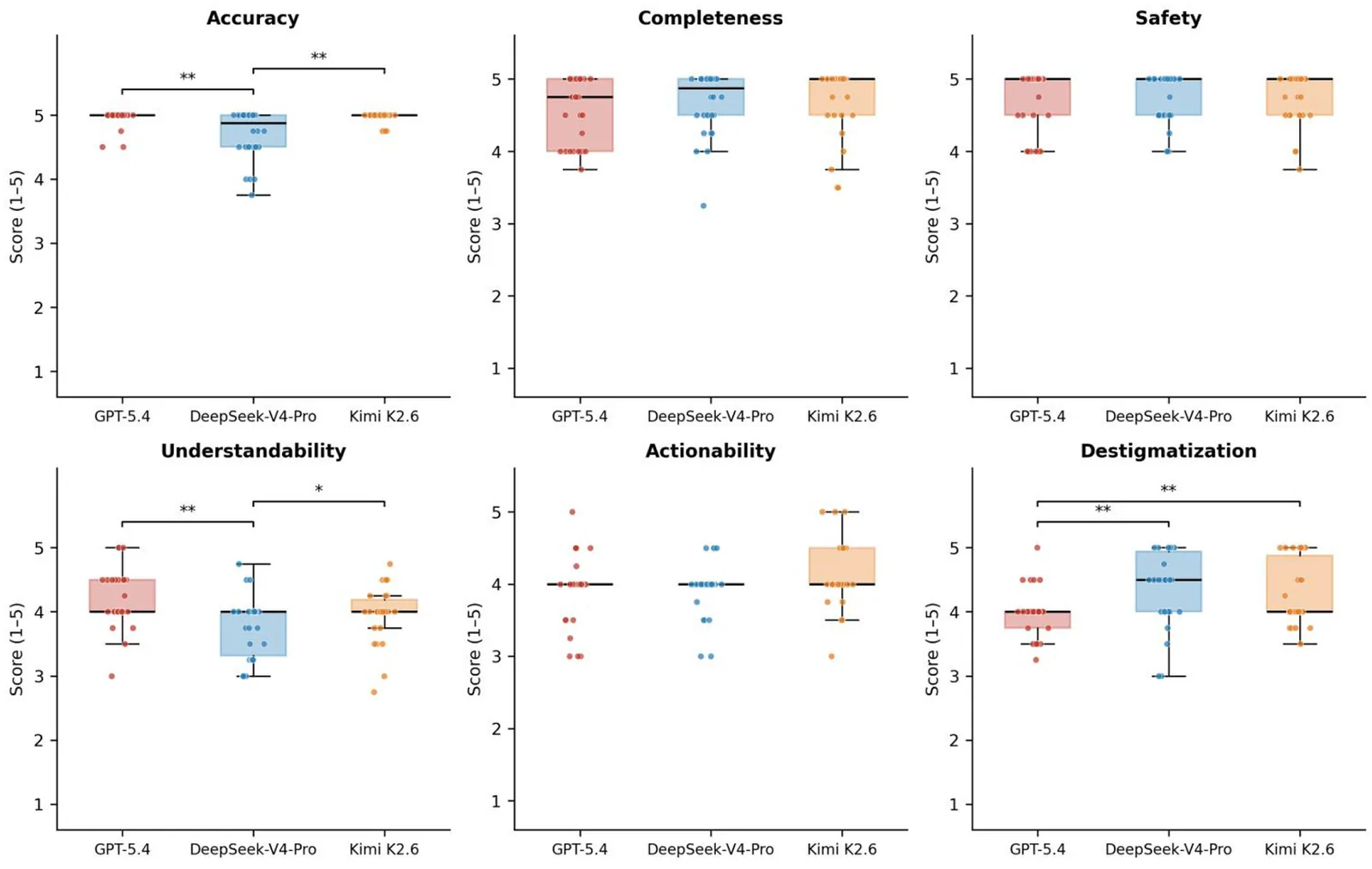
Distribution of quality scores across six evaluation dimensions by LLM. Box plots (2 × 3 panels) show the median and interquartile range, with overlaid individual query-level scores. *N* = 30 queries per model. *P < 0.05, **P < 0.01 (pairwise contrasts from the primary linear mixed-effects models with Bonferroni correction; see Table 3).

In the primary mixed-effects analysis retaining all 360 ratings per dimension, significant platform effects were detected for Accuracy (likelihood-ratio χ^2^(2) = 19.12, Holm-adjusted *P* < 0.001), Understandability (χ^2^(2) = 13.54, adjusted *P* = 0.006), and Destigmatization (χ^2^(2) = 9.16, adjusted *P* = 0.041), but not for Completeness, Safety, or Actionability (all adjusted *P* = 1.00; Table 3; Figure 2). Accuracy: GPT-5.4 and Kimi K2.6 both achieved a median score of 5.00 (IQR 5.00–5.00), while DeepSeek-V4-Pro scored lower (median 4.88, IQR 4.50–5.00). Pairwise contrasts showed that GPT-5.4 (*β* = 0.26, Bonferroni-adjusted *P* < 0.001) and Kimi K2.6 (*β* = 0.27, adjusted *P* < 0.001) both scored significantly higher than DeepSeek-V4-Pro, with no significant difference between GPT-5.4 and Kimi K2.6 (*β* = −0.01, adjusted *P* = 1.00). Understandability: all three platforms shared an identical median of 4.00, yet within-query rank patterns differed systematically: GPT-5.4 scored significantly higher than DeepSeek-V4-Pro (*β* = 0.43, adjusted *P* < 0.001), whereas the contrasts between Kimi K2.6 and DeepSeek-V4-Pro (*β* = 0.21, adjusted *P* = 0.079) and between GPT-5.4 and Kimi K2.6 (*β* = 0.22, adjusted *P* = 0.063) did not reach significance after correction. The omnibus signal is therefore driven primarily by systematically lower within-query ranks for DeepSeek-V4-Pro rather than by differences in medians. Destigmatization: DeepSeek-V4-Pro (median 4.50, IQR 4.00–4.94) and Kimi K2.6 (median 4.00, IQR 4.00–4.88) both scored significantly higher than GPT-5.4 (median 4.00, IQR 3.75–4.00; *β* = 0.38 and *β* = 0.32 vs. GPT-5.4, adjusted *P* < 0.001 and *P* = 0.006, respectively), with no significant difference between DeepSeek-V4-Pro and Kimi K2.6 (*β* = 0.06, adjusted *P* = 1.00). Completeness, Safety, and Actionability showed no significant platform differences; Safety exhibited a pronounced ceiling effect (median 5.00 on all platforms), which limits the power of both rank-based and likelihood-based tests for these dimensions. The sensitivity analysis using the original double-median aggregation with Friedman tests identified the same three significant dimensions and the same non-significant ones (Supplementary Table S6), indicating that the primary findings do not depend on the method of score aggregation.

**Table 3:** Comparison of quality scores across six dimensions: descriptive statistics [median (IQR), query-level] and primary mixed-effects omnibus results (likelihood-ratio χ^2^ with Holm-adjusted *P* across the six-dimension family).

Dimension	GPT-5.4	DeepSeek-V4-Pro	Kimi K2.6	LRT χ^2^(2)	*P* (Holm)	Kendall's W^a^	Significant contrasts^b^
Accuracy	5.00 (5.00–5.00)	4.88 (4.50–5.00)	5.00 (5.00–5.00)	19.121	< 0.001	0.286	GPT-5.4 > DeepSeek-V4-Pro (*P* < 0.001); Kimi K2.6 > DeepSeek-V4-Pro (*P* < 0.001)
Completeness	4.75 (4.00–5.00)	4.88 (4.50–5.00)	5.00 (4.50–5.00)	0.000	1.000	0.060	ns
Safety	5.00 (4.50–5.00)	5.00 (4.50–5.00)	5.00 (4.50–5.00)	0.000	1.000	0.011	ns
Understandability	4.00 (4.00–4.50)	4.00 (3.31–4.00)	4.00 (4.00–4.19)	13.540	0.006	0.267	GPT-5.4 > DeepSeek-V4-Pro (*P* < 0.001)
Actionability	4.00 (4.00–4.00)	4.00 (4.00–4.00)	4.00 (4.00–4.50)	0.000	1.000	0.058	ns
Destigmatization	4.00 (3.75–4.00)	4.50 (4.00–4.94)	4.00 (4.00–4.88)	9.163	0.041	0.245	DeepSeek-V4-Pro > GPT-5.4 (*P* < 0.001); Kimi K2.6 > GPT-5.4 (*P* = 0.006)

^a^Kendall's W is reported from the aggregated Friedman sensitivity analysis (full results in Supplementary Table S6).

^b^Pairwise platform contrasts estimated from the linear mixed-effects model with Bonferroni adjustment (three contrasts per dimension); only significant contrasts are shown.

### 3.3 Readability profiles

**Figure 3:**
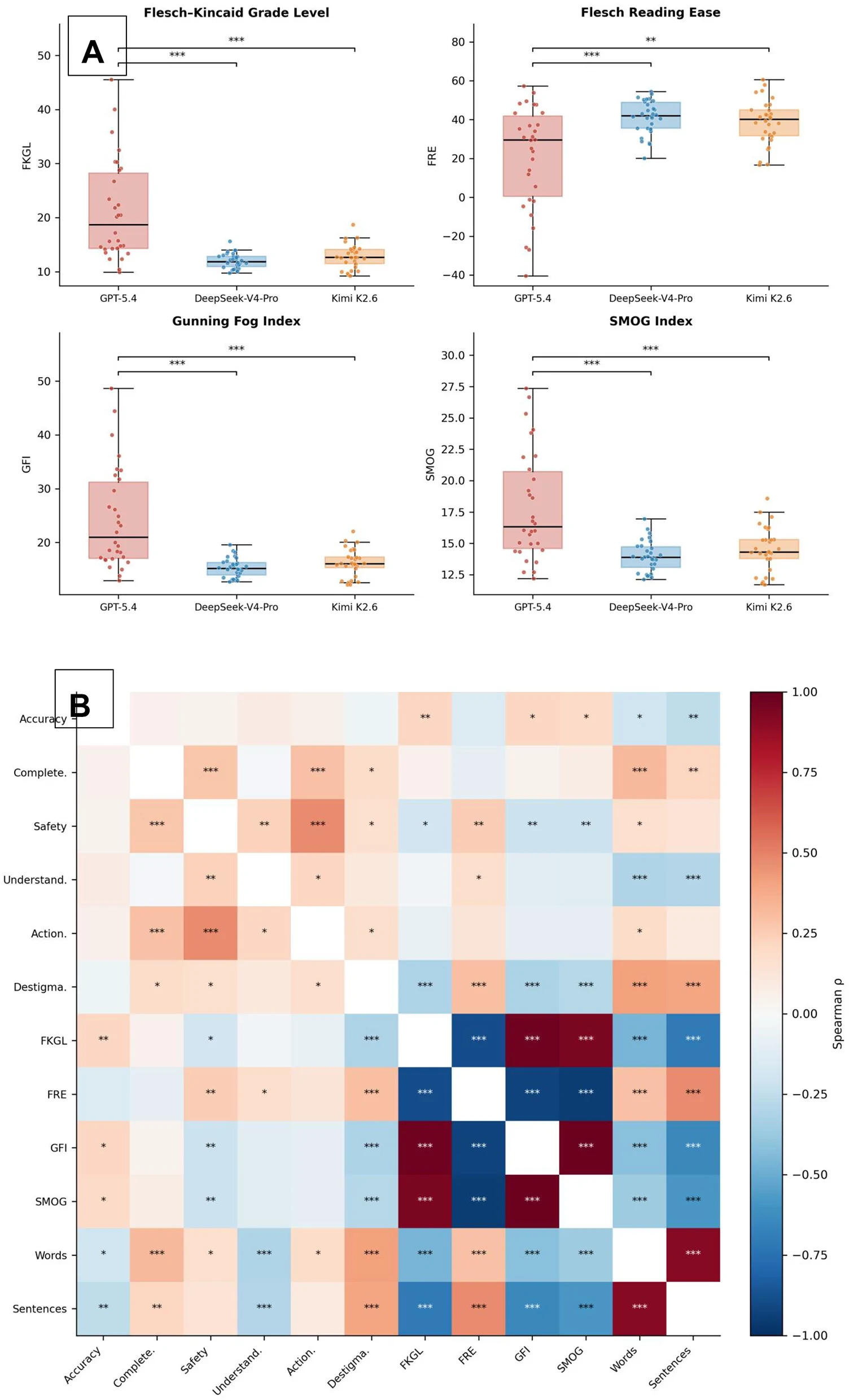
**(A)** Readability profiles across the four metrics [FKGL, FRE (higher = easier), GFI, SMOG], shown as separate panels per metric because the metrics have different numerical ranges and directions; boxes show the median and interquartile range, and points show individual query-level means (*N* = 30 per model per metric); asterisks denote Bonferroni-adjusted pairwise Wilcoxon signed-rank post-hoc contrasts (only significant contrasts are shown; **P < 0.01, ***P < 0.001; see Table 4). **(B)** Exploratory Spearman correlation heatmap among quality dimensions, readability metrics, and text-length variables (*N* = 180 responses); asterisks denote significance after Benjamini–Hochberg FDR adjustment across the 66 pairs (*P < 0.05, **P < 0.01, ***P < 0.001), with query-level cluster-bootstrap validation. The heatmap is descriptive; inferential statements in the text rely on the adjusted analysis in Supplementary Table S7.

All four readability metrics differed significantly across platforms in query-level analyses (Friedman tests, all Holm-adjusted *P* < 0.01; Table 4). GPT-5.4 produced the most difficult text (FKGL median 16.12, IQR 13.54–25.63; FRE 30.78, IQR 4.48–43.71; GFI 18.82, IQR 16.49–28.94; SMOG 15.86, IQR 14.28–21.53) and was significantly harder to read than both DeepSeek-V4-Pro and Kimi K2.6 on every metric (all Bonferroni-adjusted *P* < 0.01). DeepSeek-V4-Pro (FKGL 11.88, IQR 10.91–12.94; FRE 41.97) and Kimi K2.6 (FKGL 12.60, IQR 11.14–14.30; FRE 40.14) did not differ significantly from each other on any metric (all adjusted *P* > 0.07). All platforms remained well-above the recommended sixth-grade reading level (FKGL ≤ 6). Figure 3A visualizes these profiles in separate panels for each metric.

**Table 4:** Readability metrics by platform: descriptive statistics [median (IQR), *N* = 60 responses per model] and query-level Friedman tests (*N* = 30 matched observations per model, with Holm-adjusted *P* across the four-metric family).

Metric	GPT-5.4	DeepSeek-V4-Pro	Kimi K2.6	χ^2^(2)	*P* (Holm)	Significant contrasts^a^
FKGL	16.12 (13.54–25.63)	11.88 (10.91–12.94)	12.60 (11.14–14.30)	34.400	< 0.001	GPT-5.4 > DeepSeek-V4-Pro (*P* < 0.001); GPT-5.4 > Kimi K2.6 (*P* < 0.001)
FRE	30.78 (4.48–43.71)	41.97 (34.97–47.89)	40.14 (31.20–47.34)	9.800	0.007	GPT-5.4 < DeepSeek-V4-Pro (*P* < 0.001); GPT-5.4 < Kimi K2.6 (*P* = 0.006)
GFI	18.82 (16.49–28.94)	15.11 (13.97–16.41)	16.19 (13.75–17.97)	32.067	< 0.001	GPT-5.4 > DeepSeek-V4-Pro (*P* < 0.001); GPT-5.4 > Kimi K2.6 (*P* < 0.001)
SMOG	15.86 (14.28–21.53)	13.86 (13.05–14.63)	14.62 (12.94–15.71)	20.867	< 0.001	GPT-5.4 > DeepSeek-V4-Pro (*P* < 0.001); GPT-5.4 > Kimi K2.6 (*P* < 0.001)

^a^Bonferroni-adjusted Wilcoxon signed-rank post-hoc tests on query-level means; for FRE, lower values indicate more difficult text. DeepSeek-V4-Pro and Kimi K2.6 did not differ significantly on any metric (all adjusted *P* > 0.07).

### 3.4 Exploratory correlations

Exploratory Spearman correlations among the quality dimensions, readability metrics, and text-length variables—with query-level cluster-bootstrap CIs and FDR adjustment—are summarized in Figure 3B and reported in full in Supplementary Table S7. Of the 66 pairwise correlations, 39 remained significant after FDR control. The three grade-level indices were highly intercorrelated (ρ = 0.94–0.98); because FKGL, GFI, and SMOG share word- and sentence-complexity inputs, and because grade-level indices are partly mathematical functions of word and sentence counts, these associations are expected structural relationships rather than independent empirical findings. At the pooled level, Accuracy correlated negatively with word count (*ρ* = −0.20, FDR-adjusted *P* = 0.019) and with sentence count (*ρ* = −0.26, adjusted *P* = 0.002), while Destigmatization correlated positively with word count (*ρ* = 0.41, adjusted *P* < 0.001). Within-platform analyses, however, showed that the pooled Accuracy—word-count association was driven by between-platform differences: the correlation was +0.27 within GPT-5.4, −0.04 within DeepSeek-V4-Pro, and −0.13 within Kimi K2.6. Similarly, the Destigmatization—word-count association was strongest within DeepSeek-V4-Pro (*ρ* = 0.36). Destigmatization also correlated negatively with the grade-level readability indices (*ρ* = −0.28 to −0.32, all FDR-significant), indicating that responses using more destigmatizing language tended to be easier to read. Given the exploratory and correlational nature of this analysis, these patterns are reported as hypothesis-generating, and no causal or mechanistic interpretation is drawn.

### 3.5 Sensitivity and stability analyses

The sensitivity analysis comparing GPT-5.4 and DeepSeek-V4-Pro under uniform settings (temperature 0.7 for both) confirmed the robustness of the primary findings: Accuracy, Understandability, and Destigmatization remained significantly different, while Completeness, Safety, and Actionability remained non-significant (Supplementary Table S1). Cross-replicate stability was modest: inter-replicate ICC values, reported here without truncation, ranged from −0.05 (GPT-5.4 Accuracy, 95% CI −0.40 to 0.31) to 0.68 (DeepSeek-V4-Pro Understandability, 95% CI 0.42–0.83; Supplementary Table S3). Negative and near-zero estimates reflect a combination of pronounced ceiling effects—minimal between-query variance when most responses cluster at the top of the scale—and genuine replicate-to-replicate variability in model outputs; with only two replicates per query–platform pair, these two sources cannot be disentangled, and cross-replicate stability is therefore treated as a limitation of the study rather than attributed solely to ceiling effects.

## 4 Discussion

### 4.1 Summary of principal findings

To our knowledge, this study is the first to operationalize destigmatization as a quantifiable—though still exploratory—dimension in LLM health-information evaluation, and the first to benchmark two Chinese-developed models alongside a Western-developed frontier model for STI-related digital communication. We evaluated the quality, safety, readability, and use of destigmatizing language in STI-related responses generated by GPT-5.4, DeepSeek-V4-Pro, and Kimi K2.6. Three principal findings emerged. First, significant inter-platform differences were detected in Accuracy, Understandability, and Destigmatization, and these were robust to the analytic approach: a mixed-effects framework retaining all individual ratings and the original aggregated non-parametric pipeline identified the same three dimensions. Completeness, Safety, and Actionability showed pronounced ceiling effects with near-universal top scores. Second, the platforms exhibited distinct performance profiles rather than a uniform quality–readability trade-off: GPT-5.4 and Kimi K2.6 achieved the highest accuracy (both median 5.00, with no significant difference between them), yet GPT-5.4 produced the most difficult-to-read text, whereas Kimi K2.6 combined top-tier accuracy with significantly better readability—demonstrating that high accuracy and accessible language can coexist. DeepSeek-V4-Pro produced more readable text but scored significantly lower in accuracy, so its readability advantage cannot be described as coming without a cost to clinical correctness. Third, both Chinese-developed models used more destigmatizing language than GPT-5.4, with no significant difference between DeepSeek-V4-Pro and Kimi K2.6. Inter-rater reliability was good to excellent across all dimensions, supporting the internal consistency of the scoring process.

### 4.2 Quality dimension performance in context

GPT-5.4 and Kimi K2.6 achieved the highest accuracy scores (both median 5.00), consistent with prior evaluations that have documented strong factual performance of frontier models in clinical question-answering tasks (10, 12). However, GPT-54′s accuracy was accompanied by substantially poorer readability: its responses required a median college-level reading proficiency (FKGL 16.12), far exceeding the sixth-grade benchmark recommended for patient-education materials. This finding aligns with a growing body of evidence indicating that LLM-generated health information frequently fails to meet readability standards: evaluations of ChatGPT-4 and Gemini in patient-education contexts have documented similarly elevated grade levels (11), and a meta-narrative review of online health information demonstrated that excessively complex text is a major barrier to comprehension and health literacy, particularly among vulnerable populations (13). The pooled negative correlation between Accuracy and word count should not be read as evidence that GPT-54′s accuracy derives from conciseness: within-platform analyses showed no consistent negative association (the correlation was positive within GPT-5.4), indicating that the pooled pattern reflects between-platform differences in both accuracy and verbosity rather than a general conciseness–accuracy mechanism. For Understandability, the identical medians (4.00 across platforms) conceal meaningful distributional differences: the significant omnibus result arises from within-query rank patterns—DeepSeek-V4-Pro received systematically lower ranks across queries—rather than from differences in central tendency. DeepSeek-V4-Pro also exhibited the widest interquartile range (3.31–4.00), indicating more variable, query-dependent performance, whereas Kimi K2.6 showed the tightest distribution (4.00–4.19).

### 4.3 Destigmatization: a novel and critical dimension

The most novel finding concerned destigmatizing language: DeepSeek-V4-Pro (median 4.50) and Kimi K2.6 both scored significantly higher than GPT-5.4, with no significant difference between the two Chinese-developed models. This dimension was introduced to address a documented gap in LLM evaluation frameworks: while stigma is recognized as a major barrier to STI care-seeking and treatment adherence (14), its linguistic manifestation in AI-generated content had not previously been quantified. Recent evidence underscores why this dimension matters for sexual-health communication: sexual stigma, stress, and privacy concerns shape people's comfort in disclosing sensitive sexual-health information to AI chatbots (8), and perceived empathy and autonomy influence disclosure intentions through trust and privacy concerns (9). The tone and relational framing of AI responses may therefore influence not only immediate satisfaction but also longer-term trust in, and reliance on, AI systems for stigmatized health concerns (27, 28). The positive correlation between Destigmatization and word count (pooled *ρ* = 0.41; strongest within DeepSeek-V4-Pro) suggests that stigma-reducing language tends to require additional textual elaboration—for example, contextualizing behavior, avoiding blame-oriented phrasing, and explicitly normalizing the condition—salthough this association is correlational and partly platform-dependent. Whether DeepSeek-V4-Pro's performance reflects properties of its training corpora or deliberate alignment choices by its developers cannot be determined from our data: we did not examine training data, and all prompts were submitted in English, so any such explanation remains an untested hypothesis. We also emphasize that destigmatization was measured as an exploratory, expert-rated construct scored by two clinicians. Patients, members of affected communities, stigma researchers, and health-communication specialists were not involved in its development or scoring, and their experience of judgmental or culturally insensitive wording may differ from clinical experts' assessments. Content validation with these groups is a necessary next step before this dimension can be treated as a validated measure.

### 4.4 Readability profiles and health equity

All three platforms produced text well-above the recommended sixth-grade reading level, indicating that current LLM-generated STI information is likely to be difficult for many general readers, particularly those with limited health literacy; actual comprehension, however, was not tested among patients or lower-literacy users in this study. GPT-54′s responses were particularly challenging, with a median FKGL of 16.12 and FRE of 30.78—both indicative of difficult material. By comparison, DeepSeek-V4-Pro (FKGL 11.88) and Kimi K2.6 (FKGL 12.60) were substantially more readable, though still far from the recommended threshold. This pattern is consistent with evaluations of online health information across diverse medical domains, where academic and institutional sources routinely exceed average adult reading levels (29). The high intercorrelation among FKGL, GFI, and SMOG (*ρ* = 0.94–0.98) is expected, as these metrics share word- and sentence-complexity inputs, and should be read as convergent measurement of a shared construct of textual difficulty rather than as independent confirmation. Notably, automated readability and clinician-rated understandability did not coincide: GPT-5.4 produced the most difficult text by formula-based metrics yet received systematically higher understandability ratings from clinicians than DeepSeek-V4-Pro. This divergence suggests that the two capture different constructs—formula-based metrics index surface linguistic complexity, whereas expert raters additionally weigh structure, explanatory quality, and terminology handling—and it reinforces that neither can substitute for comprehension testing with actual patients.

From a health-equity perspective—which we present as a broader implication rather than an outcome directly evaluated in this study—the readability gap is especially concerning for STI communication. Populations most affected by STIs–including adolescents, marginalized communities, and individuals with lower educational attainment—are also those most likely to rely on digital health information and least likely to possess advanced health literacy. If AI-generated content remains difficult to read for these groups, the digital transformation of sexual health communication risks widening, rather than narrowing, health disparities. Empirical work with patient and community samples is needed to test whether the differences observed here translate into differential comprehension, trust, and care-seeking.

### 4.5 Cross-replicate stability and methodological considerations

Cross-replicate ICC values were generally modest, ranging from −0.05 to 0.68 when reported without truncation. Although negative and near-zero ICC estimates are mathematically expected when between-query variance approaches zero under Likert ceiling effects, they cannot be attributed to ceiling effects alone: with only two replicates per query–platform pair, genuine output variability and variance compression cannot be disentangled. Low inter-replicate reliability therefore also signals that the stability of responses to the same question from the same model may be limited, and single-shot assessments may miss meaningful query-dependent variability. Future studies should consider increasing the number of replicates and incorporating adversarial or edge-case queries to expand score variance and improve stability estimation.

### 4.6 Strengths and limitations

This study has several strengths. The standardized query library, informed by real-world search trends and clinical guidelines, was designed to reflect common patient information needs. Blinded, independent evaluation by two experienced dermatovenereologists yielded good to excellent inter-rater reliability (ICC(2, 1) 0.79–0.95). The primary mixed-effects analysis retained all individual ratings and accounted for the nested data structure, with convergent results from a non-parametric sensitivity analysis. The incorporation of an exploratory destigmatization dimension addresses a gap in existing evaluation frameworks and aligns with the WHO's emphasis on health equity and stigma reduction in STI control (2).

Several limitations must be acknowledged. First, the evaluation was limited to English-language, single-turn, standardized queries; real users often employ incomplete, emotional, indirect, or region-specific language and engage in multi-turn dialogue, so the findings cannot be assumed to generalize to natural interactions or to actual patient needs. Second, no patients, public representatives, or members of affected communities participated in query review, scoring, or outcome evaluation; the findings regarding understandability, destigmatization, and readability are expert judgments and may not reflect comprehension or perceived stigma among the populations most affected. Third, only two clinical raters were employed; although inter-rater reliability was good to excellent, additional and more diverse raters would further strengthen the generalizability of the scoring framework. Fourth, the destigmatization dimension is an exploratory, expert-rated construct without formal content validation, and the pilot calibration session covered only three responses. Fifth, only two replicates were collected per query–platform pair, and inter-replicate reliability was modest, limiting conclusions about output stability. Sixth, blinding was to platform labels only; models may have been recognizable to raters through formatting, disclaimer language, or writing style. Seventh, clinical content was judged primarily against US-based references (CDC guidelines), and context-dependent answers were scored against the US clinical and legal context; applicability to other health systems requires further study. Eighth, with one Western-developed and two Chinese-developed models, platform origin is confounded with architecture, training data, alignment, and configuration; the findings characterize three specific models and cannot support broader conclusions about Chinese- vs. Western-developed models. Ninth, the cross-sectional design precludes assessment of how model performance evolves with updates and retraining, and API behavior may change over time. Tenth, a small number of responses were truncated at the 2,048-token limit and were scored as generated, which may have lowered completeness-related scores for the affected responses. Eleventh, Kimi K2.6 was evaluated at temperature 1.0 (an API restriction) vs. 0.7 for the other platforms; although a uniform-setting sensitivity analysis supported the primary findings, configuration differences cannot be fully excluded. Finally, the correlation analyses, although adjusted for clustering and multiple testing, remain exploratory and do not support causal interpretation.

### 4.7 Implications and future directions

These findings carry implications for three stakeholder groups. For clinicians, the results suggest that while LLMs can provide accurate and safe STI information, the outputs should not be directly forwarded to patients without readability adaptation. For AI developers, the study highlights the need for explicit optimization of both accuracy and accessibility, as well as the incorporation of stigma-aware training objectives. For public health stakeholders, the results support the development of quality standards and continued monitoring of AI-generated health content—including readability- and language-related criteria for sensitive conditions. Because this study did not examine regulation, governance models, patient harm, or policy implementation directly, specific regulatory recommendations are beyond its scope; our findings can nonetheless contribute to the evidence base for future policy and validation studies.

Future research should extend this framework to Chinese-language and other multilingual queries, to multi-turn and ecologically naturalistic interactions, and to additional models, including open-weight variants and domain-specific medical LLMs. Formal content validation of the destigmatization dimension—with patients, affected communities, stigma researchers, and health-communication specialists—is a priority. The development of automated readability-adjustment pipelines—wherein LLM outputs are dynamically simplified to target grade levels while preserving clinical accuracy—represents a promising technical direction. Additionally, the integration of patient perspectives, rather than expert-only evaluation, would complement the present findings and ensure that readability and destigmatization improvements align with lived experience.

## 5 Conclusion

In conclusion, the three evaluated LLMs exhibited distinct profiles for digital STI health communication. GPT-5.4 and Kimi K2.6 achieved the highest accuracy, whereas GPT-5.4 produced the most difficult-to-read outputs, which are likely challenging for many general readers. DeepSeek-V4-Pro offered the most readable text but at significantly lower accuracy, and both DeepSeek-V4-Pro and Kimi K2.6 used more destigmatizing language than GPT-5.4, with no significant difference between the two. Kimi K2.6 demonstrated that high accuracy and accessible language can coexist. To our knowledge, this is the first study to quantify destigmatizing language as an explicit evaluation dimension for LLM-generated STI content, albeit as an exploratory measure pending formal content validation. Within the limits of 30 standardized English queries and three specific models, these findings can inform quality standards and continued monitoring for safer, more equitable AI-powered sexual health information systems, and they highlight the need to incorporate stigma reduction and health literacy as core design objectives in medical LLM development.

## Data Availability

The original contributions presented in the study are included in the article/Supplementary material, further inquiries can be directed to the corresponding author.
